# Developing the Strategy to Use Silk Spheres for Efficient, Targeted Delivery of Oligonucleotide Therapeutics to Cancer Cells

**DOI:** 10.2147/IJN.S519906

**Published:** 2025-06-23

**Authors:** Sara Molenda, Tomasz Deptuch, Agata Sikorska, Patryk Lorenc, Maciej Jerzy Smialek, Anna Florczak-Substyk, Piotr Pawlak, Hanna Dams-Kozlowska

**Affiliations:** 1 Department of Cancer Immunology, Poznan University of Medical Sciences, Poznan, Poland; 2 Department of Diagnostics and Cancer Immunology, Greater Poland Cancer Centre, Poznan, Poland; 3 Doctoral School, Poznan University of Medical Sciences, Poznan, Poland; 4 Department of Genetics and Animal Breeding, Poznan University of Life Sciences, Poznan, Poland

**Keywords:** bioengineered spider silk, spheres, siRNA-delivery, targeted delivery, cancer therapy, STAT3

## Abstract

**Introduction:**

Oligonucleotide-based drugs, such as siRNA, hold great promise for disease treatment, including cancer. However, their clinical application has challenges related to cell-specific delivery and susceptibility to degradation. The use of drug delivery systems (DDS) may address these problems. Nanoparticles of bioengineered spider silk demonstrate significant potential as DDS due to their biocompatibility and biodegradability. Another advantage of this material is the possibility of functionalization, which allows the control of its property. The main objective of this study was to develop a strategy for targeted delivery of oligonucleotide-based therapeutics into cancer cells using bioengineered silk technology.

**Materials and Methods:**

Two spider silk spheres that bind oligonucleotides and target cancer cells that overexpress HER2 (HER2+) were constructed. One type of sphere was made of a newly designed silk, H2.1MS1KN, which contained two functional peptides: H2.1 for binding HER2 and KN for binding oligonucleotide. The second type of sphere was formed of a blend of two previously described proteins, H2.1MS1 and MS2KN; these proteins differed not only in the functional domain (H2.1 vs KN) but also in the sequence of silk (MS1 vs MS2). The ability of proteins to bind oligonucleotides was analyzed via gel electrophoresis. The biophysicochemical properties of particles were analyzed using an SEM, NanoSight, ZetaSizer, flow cytometry, and scanning confocal microscopy. The silk particle potential was analyzed using siRNA for silencing *STAT3* expression in the HER2+ breast cancer model.

**Results:**

Both H2.1MS1KN and H2.1MS1:MS2KN proteins efficiently bound nucleic acid. H2.1MS1:MS2KN formed smaller spheres than H2.1MS1KN. Although both H2.1MS1KN and blended H2.1MS1:MS2KN spheres were effectively loaded with oligonucleotides, only H2.1MS1:MS2KN spheres delivered siRNA to HER2+ cancer cells that successfully silenced *STAT3* expression.

**Conclusion:**

Not only the selection of functional peptides but also their quantity and type of silk is crucial when developing an effective silk-based DDS for delivering active siRNA.

## Introduction

Cancers are currently one of the most significant challenges in modern medicine. It is treated using various methods, including surgery with chemotherapy and/or radiation therapy, immunotherapy, targeted therapy, or hormone therapy.^1^ Recently, one of the rapidly advancing methods is gene therapy, which involves controlling the expression of genes that play a crucial role in the development and progression of cancer.^2^ Targeting *STAT3* using oligonucleotides such as siRNA, shRNA, or ASO is an example of this type of therapy.^3–8^ The molecule siRNA *STAT3* (si*STAT3*) silenced *STAT3* expression leading to the induction of apoptosis and the inhibition of the proliferation and migration of breast cancer cells^9^,^10^ indicating its potential as therapeutics.

Nevertheless, the gene therapy faces considerable challenges. Due to the therapeutic mechanism, most nucleic acid-based therapeutics must be delivered inside the cells. The large size and negative charge of oligonucleotide therapeutics hinder their ability to penetrate cell membranes effectively.^11^ Another limitation is that RNAses and DNAses rapidly degrade nucleic acid-based therapeutics when applied in vivo.^12^ A further challenge is that nucleic acid-based therapeutics are target-specific (regarding mRNA sequence) but not selective for the type of cells they should affect.^13^

Some limitations associated with applying oligonucleotide-based drugs can be overcome through chemical modifications such as adding phosphorothioate (PS), locked and unlocked nucleotides, and substituting the ribose 2’-OH group.^4^,^14–16^ Another approach is using a Drug Delivery System (DDS); however, a highly effective DDS should meet several essential criteria. It should be capable of specifically targeting and interacting with the intended cell type, even within a complex tissue environment. Moreover, DDS should be internalized by cells via endocytosis, allowing the drug to escape from the endosome, and its transport functions must be carried out without causing an adverse toxic and immunological response. Desirable is also that the carrier is biodegradable. It should also exhibit appropriate particle size and stability.^11^,^17^

DDS has already shown numerous advantages for drug application, including reducing the systemic side effects of medication, enhancing the efficiency of drug delivery, enabling controlled release, and improving the drug’s specificity.^18^ DDS based on different kinds of materials were tested in the delivery of various types of therapeutics, such as small molecule drugs, chemotherapeutics, gene therapy agents, antibodies, or enzymes.^19–21^ Despite the numerous advantages associated with the use of drug delivery systems, they also face certain limitations and challenges. Most DDS utilize the Enhanced Permeability and Retention (EPR) effect to deliver drugs to tumors. However, the limited specificity of this approach often leads to drug toxicity in healthy cells. As a result, there is a growing focus on developing DDS that can selectively target cancer cells or their specific components/characteristics while reducing the exposure of healthy cells to the drugs.^22^,^23^ Further challenges associated with the use of drug delivery systems include low bioavailability, limited drug loading capacity, and stability issues.^24^ Another limitation is that many drug delivery systems require complex and costly manufacturing processes.^25^ DDS may require expensive components or complex methods for their production or modification.

Drug delivery systems (DDS) are increasingly being applied in clinical settings.^26^ Currently, many therapeutics are administered using dedicated carriers. Nowadays, highly toxic drugs used in cancer treatment, such as doxorubicin (Doxil) and paclitaxel (Abraxane or Pazenir), may be delivered to tumors through drug delivery systems.^27^,^28^ This paclitaxel delivery method significantly reduces its drawbacks, increasing its stability, blood circulation time, and tumor uptake.^29^,^30^ However, nanomedicines face significant limitations, including high production costs, lengthy commercialization timelines, and complex manufacturing challenges, particularly for biologically based formulations. Clinical translation is also hindered by regulatory delays and inconsistent toxicological data, limiting their broader adoption.^29^

DDS for oligonucleotide-based therapeutics can be based on viral and non-viral carriers.^31^ Currently, non-viral systems are considered more promising.^32^ Polymeric, lipid-based, or inorganic nanostructures are most commonly utilized.^21^,^33^,^34^ These nanostructures can be functionalized with aptamers, peptides or antibodies to provide specificity toward target cells.^35^,^36^

Spider silk is regarded as a material with significant potential for biomedical applications, including cancer treatment.^37^ Silk can be obtained from nature, but the procedure is inefficient.^38^ Spider breeding is impossible due to their territorial and cannibalistic behavior. Moreover, silk harvested from nature can be a mixture of several types of spider silk proteins, which is inappropriate for some application (including medical) due to the problem of lack of reproducibility. Therefore, bioengineered silk was proposed as a more applicable material due to control over silk composition, purity, and reproducibility. Recombinant spider silk proteins were used to produce various biomaterials, including drug carriers, films, foams, and scaffolds.^39–41^ The process of obtaining recombinant spider silk protein begins with constructing a synthetic gene encoding the desired silk. The frequency of codon sequences occurring in the new gene is consistent with the codon frequency occurring in the future host, which can improve protein production yield. The prepared expression vector containing a gene encoding bioengineered silk is then inserted into the chosen expression system. The bacterial expression system is the most frequently employed for producing recombinant silk proteins due to its relatively simple production, rapid bacterial generation time, cost-effectiveness, and scalability of the process, which are key advantages of this method.^42^,^43^

An additional advantage of constructing synthetic genes of bioengineered proteins is the ability to introduce sequences that encode functional peptides.^42^ Such DNA modification results in each protein molecule containing a functional peptide. The functional peptides can modify the properties of the carrier and influence tumor targeting, cellular binding, or inorganic molecules binding.^42^,^44^ Various studies have shown that modified spider silk spheres can be efficient drug delivery carriers.^40^,^45–49^

In addition to the wide range of possibilities for producing different variants of bioengineered silk, the silk blending strategy broadens its potential applications. To generate more advanced materials with multiple functions, two types of silk designed for different purposes can be combined.^37^ In such strategy, silk functionalized to enhance its specificity to target cell can be blended with silk functionalized with a peptide that control drug-loading.^50^,^51^ Moreover, blending two types of silk can modify the physicochemical property of spheres.^52^

Our team previously demonstrated that spheres made of functionalized spider silk can effectively deliver doxorubicin to the HER2-overexpressing cancer cells.^53^ The human epidermal growth factor receptor 2 (HER2) belongs to the epidermal growth factor receptor (EGFR) family of receptor tyrosine kinases, which are situated on the cell membrane.^54^ It is not only a target for therapy itself (important clinically^55–57^) but is often used as a ligand for functionalized particles for intracellular drug delivery.^58–62^ In our system, the spider silk spheres modified with the H2.1 peptide (H2.1MS1) were effectively internalized and delivered the drug to HER2-overexpressing cancer cells.^63^ Once inside the cells, the silk spheres accumulate in lysosomes and undergo degradation; the lysosomal function is essential for silk-based carrier elimination.^63^ The therapeutic efficacy of the drug delivered by H2.1MS1 spheres was indicated in both in vitro and in vivo studies.^53^,^64^ The spheres did not cause toxic and immune adverse side effects, confirming its biocompatibility.^65–67^ We also demonstrated in another study that incorporating the poly-lysine peptide (KN) into different type of spider silk protein MS2 (MS2KN) made it an effective carrier for oligonucleotide-based drugs.^68^ Adding the KN peptide to silk significantly enhanced its ability to bind nucleic acids. Furthermore, encapsulating the oligonucleotide therapeutic within the spheres protected it from degradation by nucleases and extended its target gene silencing property.^68^ Although the oligonucleotide-loaded spheres were effectively internalized by macrophages, it should be pointed out that such system delivers oligonucleotide therapeutics to cells in a non-specific manner.

In the presented study, we generated a DDS that binds to HER2-overexpressing cancer cells through the H2.1 peptide but also effectively binds nucleic acids by KN peptide. Such DDS should specifically deliver oligonucleotide-based therapeutics to HER2+ cancer cells. We compared two systems designed to fulfill this objective. In the first system, we constructed an H2.1MS1KN silk containing both peptides to produce nanocarriers. The second strategy involved using a blend of two proteins, H2.1MS1 and MS2KN, each containing one of the desired peptides. We compared these two strategies regarding nanoparticle properties including their efficiency in binding nucleic acids and HER2-overexpressing cancer cells. Finally, we evaluated the effectiveness of *STAT3* silencing as a result of si*STAT3* delivery by both types of spheres to the HER2+ cancer cells. We indicated that not only the selection of functional peptides, but also their quantity and type of silk is crucial when developing an effective silk-based DDS for delivering active siRNA.

## Materials and Methods

### Construction of Expression Plasmids pETNX-H2.1MS1KN

MS1, H2.1MS1, and MS2KN constructs have been previously designed.^53^,^68^,^69^ The new construct H2.1MS1KN was generated by cloning an oligonucleotide sequence encoding poly-lysine to bind nucleic (KN) into the SpeI restriction site of the H2.1MS1 (13mer) construct for its 3′-terminal functionalization. The sequences of the added oligonucleotides were: KN.F:5′-CTAGCAAAAAGAAAAAAAAGAAAAAGAAAAAAAAGAAAAAGAAAAAGAAAA,KN.R:3′-GTTTTTCTTTTTTTTCTTTTTCTTTTTTTTCTTTTTCTTTTTCTTTTGACT. The KN.F and KN.R oligonucleotides were annealed to form double-stranded DNA fragments with cohesive ends complementary to those generated by NheI and SpeI. The sequence of the resulting plasmid was confirmed by sequencing at the Adam Mickiewicz University Core Facility in Poznan. The restriction enzymes and alkali phosphatase were supplied by Fermentas (Thermo Fisher Scientific Inc., Waltham, MA), while ligase by New England Biolabs, Inc. (Ipswich, MA).

### Production and Purification of Bioengineered Spider Silks

Lab-scale production of MS1, H2.1MS1, H2.1MS1KN, and MS2KN proteins was performed in a Bioflo 415 fermentor (New Brunswick Scientific, Edison, NJ). MS1 and H2.1MS1 silk proteins were purified by using the thermal method 80:20, as previously described.^69^ In brief, the bacterial pellet was resuspended in lysis buffer containing 100 mm NaCl, 20 mm HEPES (4-(2-hydroxyethyl)-1-piperazineethanesulfonic acid), pH 7.5, and Pierce™ Protease Inhibitor Tablets, EDTA-free (ThermoFisher, Waltham, MA). Lysozyme (Sigma, St. Louis, MO), 0.2 mg/mL, was added to the solution to lyse the bacteria; then the mixture was incubated at 4°C with agitation for 30 min. Additionally, the bacteria and their structures were damaged by sonication. DNA was degraded by incubation with DNaseI (Sigma, St. Louis, MO; 0.1 mg/mL) supplemented with 3 mm MgCl_2_ (Sigma, St. Louis, MO). Then, bacterial proteins were denatured at 80°C for 10 min and removed by sedimentation at 21,000 × g for 30 min at 4°C. The thermal treatment was repeated at 80°C for 20 min, followed by centrifugation as described above. Next, the spider silk protein was precipitated overnight at 4°C with 20% ammonium sulfate (VWR, West Chester, US). The next day, the protein was sedimented (30 min, 7000 × g) and washed with 20% ammonium sulfate. The precipitated silk was dissolved in 6 M guanidine thiocyanate and dialyzed using Cellulose Dialysis Tubing cutoff 14,000 Da (Sigma, St. Louis, MO) against 10 mm TRIS-HCl buffer pH 7,5- or 50 mm Sodium Borate pH 8,5 depending on further application.

To purify proteins with a nucleic acid-binding peptide (KN), the propionic acid-based method was employed as described before with a modification.^69^ Five milligrams of bacterial pellets were thoroughly disrupted in 5 mL of 13.43 N propionic acid. The resulting mixture was then diluted with 19 mL of sterile water and incubated at 4°C for one hour with agitation. The precipitated proteins and cells debris were centrifuged at 21,000 × g for 30 min, and the supernatant was collected. Next, NaCl was added to the supernatant at a final concentration of 2 M, incubated at 4°C for 1 h, and then centrifuged at 21,000 × g, for 30 min. After collection of supernatants, silk proteins were precipitated using 20% ammonium sulfate, and further steps of protein purification were processed according to the 80:20 method. The predicted molecular weight of the H2.1MS1KN protein is 38.56 kDa, and the extinction coefficient is 40340 M-1 cm-1.

All proteins underwent a rigorous analysis using the standard scientific method of SDS-PAGE gel electrophoresis. A 12.5% gel was used, and the proteins were stained with colloidal blue (Roti-Blue; Carl Roth, Karlsruhe, Germany). This method provided us with a comprehensive understanding of the proteins’ characteristics and purity.

### Nucleic Acid Binding Assay

To test the protein’s ability of nucleic acids binding, 1 µL of 50 µM siRNA *Luc* (si*Luc*) (Table 1) was mixed with H2.1MS1, H2.1MS1KN, MS2KN, or H2.1MS1:MS2KN blend (80:20) at a ratio of 1:0, 1:1, 1:10, 1:100, and 1:200, respectively. The binding was performed in 10 mm Tris-HCl pH 7.5. After 5 min of incubation at RT, complexes were analyzed in 2% agarose gel stained with 1,5% Ethidium Bromide (Sigma, St. Louis, MO). The gel was imaged using a G-box transilluminator (Syngene, Frederick, MD) and analyzed using GeneTools software (Syngene, Frederick, MD).

**Table 1:** List of siRNA Sequences Used for Treatment

Name	Sequence	Sequence
*siLuc*	5’-UCGAAGUACUCAGCGUAAGTT-3’	5’- CUUACGCUGAGUACUUCGATT −3’
*siSTAT3*	5’-CAGGGUGUCAGAUCACAUGGGCUAA-3’	5’-UUAGCCCAUGUGAUCUGACACCCUGAA-3’
iFam-si*Scr*	5’- AUGUAUUGGCCUGUAUUAGTT −3’	5’- /56-FAM/CUAACAUAGGCCAAUACAUTT – 3’

### Protein Labeling

MS1, H2.1MS1, or H2.1MSKN protein (1 mg) in 50 mm Sodium Borate, pH 8,5, was mixed with Cy^®^5 Mono NHS Ester (50 µg) (Cytiva, Danaher Corporation, Washington, DC) and incubated in the dark for 1 hour, according to manufacturer’s protocol. After incubation, the excess dye was removed using PD10 desalting columns (Cytiva, Danaher Corporation, Washington, DC) following the producer’s instructions.

### Silk Spheres Production

The spheres were formed using the micropipette method or by a micromixing device. For the blended spheres, H2.1MS1 protein [0,5 mg/mL] was mixed with MS2KN silk [0,5 mg/mL] at a ratio of 80:20. In the micropipette method, 100 µL silk solution at 0.5 mg/mL concentration was mixed by pipetting with 1000 µL of 2 M Potassium Phosphate, pH 8 (Sigma, St. Louis, MO). In the micromixing method, 1 mL of protein at a concentration of 0.5 mg/mL was mixed with 10 mL of 2 M Potassium Phosphate, pH 8, using a Nemesys ultra high-pressure syringe pump (CETONI GmbH, Korbußen, Germany). The silk and 2 M Potassium Phosphate (pH 8) solutions were delivered through tubing with a diameter of 250 nm at flow rates of 10 µL/s and 100 µL/s, respectively, and subsequently mixed at a 1:10 ratio in T-element with a diameter of 500 nm. The obtained particles were incubated in 2 M Potassium Phosphate, pH 8 overnight at room temperature. After dialyzing against ultrapure water, the spheres were centrifuged at 21,000 × g for one hour and redispersed in PBS or deionized water for further analysis.

### Loading of siRNA into Silk Spheres

Spheres loaded with siRNA were formed by mixing the silk and nucleic acid complexes with a 2 M Potassium Phosphate, pH 8. First, 50 μL of H2.1MS1KN 1 mg/mL [26µM] soluble silk protein and 50 μL of 20 μM iFam-si *Scrambled* (iFAM-si*Scr*), si*Luc*, or si*STAT3* (Table 1) were mixed and incubated at room temperature for 5 min. The following production strategy was employed for blended spheres: first, 10 μL of MS2KN 1 mg/mL [20µM] soluble silk protein and 50 μL of 20 μM iFam-si*Scr*, si*Luc*, or si*STAT3* were mixed and incubated at room temperature for 5 min. Then, 40 µL of H2.1MS 1 mg/mL [23µM] soluble silk protein was added. The complexes silk:siRNA were then used to form sphere as described at point 2.5.

For loading efficiency determination, the iFam- si*Scr* loaded spheres were used. After loading and spheres sedimentation, the supernatant was measured for fluorescence of unloaded iFam- si*Scr* using Victor X2 Multilabel Microplate Reader (PerkinElmer, Waltham, MA). A standard calibration curve for the iFam- si*Scr* was used for drug quantification. To calculate the efficiency of iFam- si*Scr* loading into spheres, the following formula was used:

$${\mathrm{loading\ efficiency}}\,\left({\mathrm{\%}}\right)\,=\,{{{\mathrm{the\ amount\ of\ drug\ added}}-{\mathrm{the\ amount\ of\ drug\ remained\ in\ supernatant}}}\over{{\mathrm{amount\ of\ drug\ added}}}}\,\times100\rm\%$$

### Characterization of Silk Spheres by Scanning Electron Microscopy (SEM)

The suspension of silk spheres in water was applied onto coverslips (Thermo Scientific/Nunc, Langenselbold, Germany) and allowed to air-dry. Samples were sputtered with gold under a vacuum in a Quorum Sputter Coater Q150T ES (Quorum Technologies, Ringmer, UK) and analyzed using SEM (JEOL JSM-7001F, JEOL Ltd., Peabody, USA) at 10 kV of accelerating voltage.

### Analysis of the Size of Silk Spheres

The sphere size was measured using two distinct methods. The first method involved analyzing images acquired through Scanning Electron Microscopy (SEM), with particle sizes determined using ImageJ 1.46r software. The size analysis was conducted based on three SEM images taken from three independent silk sphere preparation. Thirty measurements were taken from each photo. The second method utilized the NanoSight instrument (Malvern Panalytical, Malvern, GB). To measure the particle size, the spheres were suspended in 3 mL of distilled water at a concentration of 50 µg/mL and sonicated for 5 minutes in an ultrasonic water bath. The experiment was performed three times.

### Zeta Potential (ZP) Analysis of Silk Spheres

To measure the zeta potential of particles, 5 μL of spheres (1 mg/mL) were suspended in 10 mL of distilled water and sonicated for 5 min in a sonic water bath. The spheres were then analyzed using a ZetaSizer (Malvern Panalytical, Malvern, GB). The experiment was conducted three times in triplicate.

### Cell Cultures

Human HER2 overexpressing ovarian cancer cells SKOV3 (ATCC, Manassas, VA) and HER2 negative breast cancer cells MDA-MB-231 (ATCC, Manassas, VA) were used in the study. Cells were cultured at 37°C in a humidified atmosphere containing 5% CO_2_. Cells were grown in Dulbecco’s Modified Eagle Medium (DMEM; Coring, New York, NY) supplemented with 10% FBS (Biowest, Nuaillé, France) and 80 μg/mL gentamicin (KRKA, Novo Mesto, Slovenia).

### Cytotoxicity of Silk Spheres

A total of 2×10^4^ SKOV3 cells per well were seeded into a 96-well plate and incubated overnight. The following day, varying H2.1MS1KN or H2.1MS1:MS2KN silk spheres concentrations were introduced into the cell cultures. Untreated cells served as a negative control. After 3 hours, the unbound particles were removed from the medium, and the cells were cultured for 48 hours. Mitochondrial activity was then measured using the MTT assay. A solution of 3-(4,5-Dimethyl-2-thiazolyl)-2,5-diphenyl-2H-tetrazolium bromide (MTT) at a concentration of 1 mg/mL (VWR, Radnor,r, PA) was added to the cells, followed by incubation at 37°C for 4 hours. After incubation, the medium was removed, and the resulting formazan crystals were dissolved in Dimethyl Sulfoxide (DMSO) (Chempur, Piekary Slaskie, Poland). The relative cell viability (%) was calculated as (test sample/negative control) × 100%. This experiment was performed in triplicate and repeated three times.

### Cell Binding Assay of Silk Spheres

For flow cytometry analysis, SKOV3 cells were washed with PBS and detached using a non-enzymatic cell dissociation solution (Sigma, St. Louis, MO). Next, 10 µg/mL spheres labeled with Cy5 were mixed with 1×10^5^ cells suspended in PBS with BSA [1%] and incubated for one hour at 4°C in the dark. After three times washing with PBS, cells were investigated with a Navios flow cytometer (Beckman Coulter, Brea, CA) and analyzed using FlowJo Software (Becton Dickinson, Franklin Lakes, NJ).

### Cellular Uptake of Silk Spheres

The SKOV3 and MDA-MB-231 cancer cell lines were seeded at a quantity of 3×10^4^ on 8-well Lab-Tek^®^ II chambered coverglass (Nunc, Rochester, NY) 24 hours before the experiment. Next, cells were incubated for 4 hours with 5 µg/mL of Cy5-labeled spheres at 37°C, washed twice with PBS, and finally fixed with 4% paraformaldehyde (Agar Scientific, Rotherham, UK) for 10 min. After fixation, the cell membrane was stained with 50 μg/mL FITC-labeled Concanavalin A (Sigma, St. Louis, MO) for 30 min and then coated with a FluoroShield containing DAPI (Sigma, St. Louis, MO). The cells were imaged using a Confocal Laser Scanning Microscope Zeiss LSM 880 Airyscan system (Jena, Germany). Filter sets used included a 488 nm filter with band pass 491–553 nm (laser Argon2), 410–480 nm DAPI (laser Diode 405), and 543 nm with band pass 584–632 nm (laser HeNe1). Plan-Apochromat 63×/1.4 Oli DIC M27 objective was used.

### Real-Time Quantitative PCR

The SKOV3 cells were seeded at 1×10^5^ per well of a 12-well plate and cultured for 24 h. Subsequently, cells were treated with si*STAT3* delivered by spheres at a dosage of 250 nM. The spheres loaded with si*Luc* were used as a control. After 48 hours, RNA was extracted using Fenozol (A&ABiotechnology, Gdansk, Poland) according to the manufacturer’s protocol. RNA was quantified using NanoDrop (Thermo Scientific, Wilmington, DE), and 1 μg of total RNA was used for cDNA synthesis with iScript Reverse Transcription Supermix (Bio-Rad, Irvine, CA). QPCR primers specific for human STAT3 and GAPDH were designed using Primer3Plus software and are listed in Table 2. Sequence-specific amplification was performed with Maxima SYBR Green/ROX qPCR Master Mix (ThermoFisher Scientific, Waltham, MA) and LightCycler 480 (Roche, Basel, Switzerland). Data was normalized to GAPDH expression, and the relative expression levels compared to si*Luc* were calculated using the 2−ΔΔCt method. The experiment was repeated six times.

**Table 2:** List of Primer Sequences Used for qPCR

Gene	Forward Primer	Reverse Primer
*GAPDH*	5’-TCACTGCCACCCAGAAGACT- 3’	5’- ATGCCAGTGAGCTTCCCGTT- 3’
*STAT3*	5’- GGAATAACGGTGAAGGTGCT −3’	5’- CATGTCAAACGTGAGCGACT- 3’

### Statistics

To analyze the significant differences between H2.1MS1KN and H2.1MS1:MS2KN groups, for samples of normal distribution, the *t*-test was used. In cases of non-normal distribution, the Mann–Whitney test was employed to establish the level of significance between the groups. The differences were considered significant when p < 0.0001 (****), p < 0.001 (***), p < 0.01 (**), p < 0.05 (*). The data presented are expressed as mean ± standard error of the mean. The statistical differences were calculated with GraphPad Prism 8 software.

## Results and Discussion

### Production and Purification of Bioengineered Spider Silk Variants

The aim of this study was the construction of an efficient, stable, and cell-specific silk-based delivery system for therapeutic siRNA molecules to HER2-overexpressing cancer cells. Our team previously designed, produced, and purified two bioengineered silk variants, MS1 and MS2.^69^,^70^ The bioengineered silk MS1 was based on the MaSp1 protein and the MS2 silk on MaSp2 from *N. clavipes* spider. Both MS1 and MS2 proteins can be modified by the addition of short peptides, which control their properties.^45^,^50^,^52^,^68^ Previous studies have shown that spheres made of the MS1 protein functionalized with H2.1 peptide (H2.1MS1, MYWGDSHWLQYWYE) can effectively bind and internalize into HER2+ cancer cells,^53^ while the modification of MS2 silk with poly-lysine KN (K15) peptide enables it to bind nucleic acids.^68^ In this study, we examined the possibility for construction of DDS based on properties of H2.1MS1 and MS2KN for delivery oligonucleotide therapeutics into HER2+ cells. The first strategy involved adding a KN peptide into H2.1MS1 spider silk protein sequence. The second strategy involved mixing both spider silk proteins to obtain blend of protein with desired properties.

**Figure 1:**
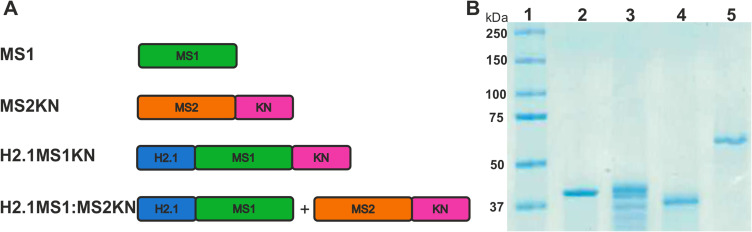
Spider silks constructs and SDS-PAGE analysis of silks. (**A**) A schematic representation of the spider silk protein constructs. Building blocks: - MS1 – MS1 silk (green), MS2 – MS2 silk (Orange), KN – nucleic acid binding peptide (pink), H2.1 – HER2 binding peptide (blue). Constructs: MS1 – control MS1 silk, MS2KN – MS2 silk functionalized with KN peptide, H2.1MS1KN - MS1 silk double-functionalized with H2.1 and KN peptides, H2.1MS1:MS2KN – blend of H2.1MS1 (MS1 silk functionalized with H2.1 peptide) and MS2KN (MS2 silk functionalized with KN peptide) silks. (**B**) Electrophoretic analysis of control and hybrid proteins using 12.5% SDS-PAGE gel. 1. Protein molecular weight marker (Precision Plus Protein™ Kaleidoscope™ Prestained Protein Standards, BioRad), 2. MS1 silk (MW 39.25 kDa), 3. H2.1MS1 silk (MW 41,68 kDa), 4. H2.1MS1KN silk (MW 38.56 kDa), and 5. MS2KN silk (MW 48,83 kDa).

The schematic representation of the spider silk protein constructs used in this study is illustrated in Figure 1A. As mentioned above, the proteins MS1, H2.1MS1 and MS2KN have been previously characterized.^53^,^68^,^69^ A novel bioengineered spider silk H2.1MS1KN was constructed by adding oligonucleotide sequence encoding KN peptide to H2.1MS1 (13mer) silk. The average yield of purified MS1 silk protein was approximately 1.5 mg, H2.1MS1 0.8 mg, MS2KN 0.6 mg, and H2.1MS1KN 0.2 mg per gram of bacterial pellets. The SDS-PAGE analysis indicated that except for the H2.1MS1 variant, silks did not show a sense of degradability, and all proteins exhibited high purity (Figure 1B). Moreover, the proteins MS1, H2.1MS1, and H2.1MS1KN migrate in accordance with expected molecular weight, however, MS2KN displayed higher molecular weight than expected (Figure 1B). Previously, we indicated that various types of MS2 silk (9-mer or 15-mer), although migrated in the SDS-PAGE gel not in accordance with expected molecular weights, the MALDI-TOF results aligned with the predicted values.^70^,^71^ One of the possible explanations for the impaired migration on the SDS-PAGE gel of the MS2-type silk is a relatively high content of proline residues (15.7%) in contrast to the MS1-type protein. It is considered that high proline content affects the conformation of the protein/SDS complex, causing low mobility on SDS-PAGE.^72^ The cyclic structure of proline imparts rigidity to the polypeptide chain, hindering the complete unfolding of the protein under SDS-PAGE conditions and altering its electrophoretic migration. Overestimation of molecular mass based on SDS-PAGE analysis has been reported for proline-rich proteins such as gliadin, hordein, secalin, calf thymus histone I, and collagen fragments.^72^

To obtain all silk variants, we applied two purification protocols. The standard method utilizes high temperature (80°C) to denature the bacterial proteins, while the soluble silk in the supernatant is then precipitated with 20% ammonium sulfate (method named 80/20). Although initially, we purified all silk variants using a thermal method, the procedure had to be changed due to the high endogenous nucleic acid contamination of H2.1MS1KN and MS2KN silks (data not shown, manuscript in preparation). The KN peptide could bind the bacterial nucleic acids, and during the standard purification protocol, the nucleic acids did not separate from silk, ultimately causing contamination. Modifying the purification protocol using an acid environment^69^ proved to be a superior method for the purification of the proteins with the KN peptide (manuscript in preparation). Shortly, the application of a high concentration of NaCl in acid condition led to the dissociation of the nucleic acids from the protein, which then allowed silk to be salted out using ammonium sulfate. The silk-purification protocol was modified based on the method used for the extraction of proteins, which are known to interact with DNA in cells, like histones.^73–75^ High salt decreases a non-specific interaction between the protein and impurities (like DNA) due to the interruption of the electrostatic interactions between them. Such an approach improved the H2.1MS1KN quality since the silk purity, expressed by the ratio of absorbance measured at 260 and 280 nm (260/280), was less than 0.6.

Additionally, the H2.1MS1KN silk demonstrated a considerably lower (four times) final yield than the H2.1MS1 protein. It could result from the double functionalization of MS1 silk, especially adding a poly-lysine peptide, which could be toxic to bacterial hosts.^76–78^

### Analysis of siRNA Binding by Soluble Silk Variants

**Figure 2:**
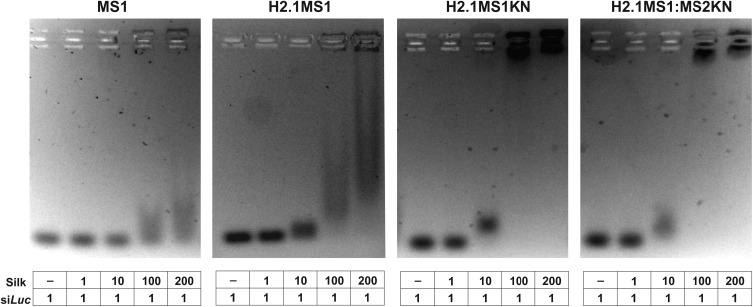
Efficiency of siRNA binding to spider silk proteins. The si*Luc* and the soluble bioengineered silks (MS1 (control), H2.1MS1 (control), H2.1MS1KN, and blend of H2.1MS1 and MS2KN (H2.1MS1:MS2KN) were mixed at different molecular ratios and incubated for 5 min at room temperature. The binding of oligonucleotides to silk proteins was analyzed using agarose gel electrophoresis.

The study aimed to construct carriers for nucleic acid-based drug delivery. Thus, we compared the silks in terms of their nucleic acid binding capabilities. The ability of MS1, H2.1MS1KN, H2.1MS1, and H2.1MS1:MS2KN soluble proteins to bind oligonucleotides was tested in agarose gel assay. As indicated in Figure 2, the MS1 silk variant cannot form complexes with si*Luc*. Although adding H2.1 peptide to MS1 silk constructs slightly increased the nucleic acid binding property, the KN peptide was the one that considerably increased the oligonucleotide binding to both tested silk variants H2.1MS1KN and the blend of H2.1MS1:MS2KN (Figure 2).

Interestingly, the H2.1 peptide increased the nucleic acid binding potential. Based on the H2.1 peptide composition, the additional negatively charged amino acids were introduced to the construct (Asp and Glu), which should decrease the oligonucleotide binding due to electrostatic repulsion. On the other hand, additional histidine (His) was also introduced, which could increase oligonucleotide attachment. However, the reaction was performed at 10 mm Tris pH 7.5, and H should be relatively neutral at such pH. Nevertheless, the most pronounced effect induced poly-lysine peptide. At the pH neutral, the positively charged Lysine (pK 10.5) effectively interacts with nucleic acid. It should be pointed out that despite only 20% content of the MS2KN protein in the H2.1MS:MS2KN blend, the oligonucleotide binding property was similar to one with silk with both functionalizations, in which each molecule possesses one KN peptide.

### The Properties of H2.1MS1KN and H2.1MS1:MS2KN Silk Spheres

**Figure 3:**
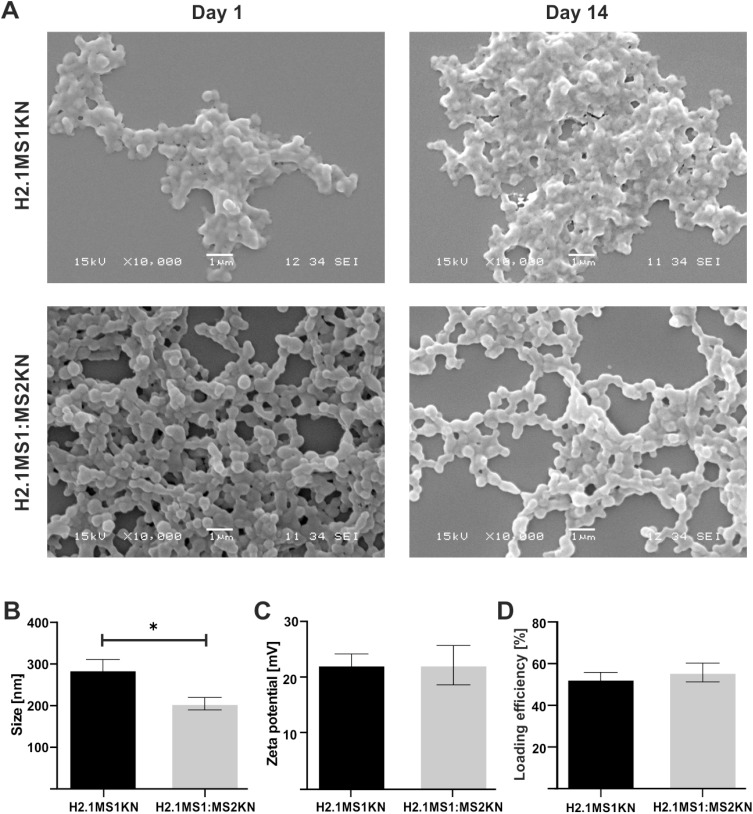
Physicochemical properties of the H2.1MS1KN and H2.1MS1:MS2KN spheres. (**A**) The morphology of H2.1MS1KN and H2.1MS1:MS2KN spheres analyzed by SEM (scale bar - 1 μm). (**B**) The size of H2.1MS1KN and H2.1MS1:MS2KN spheres (D90) was measured using NanoSight at the spheres concentration of 0.05 mg/mL (**C**) Zeta potential of H2.1MS1KN and H2.1MS1:MS2KN spheres was measured at 25 °C in ddH_2_O at the sphere concentration of 0.01 mg/mL. (**D**) Loading efficiency of iFam-si*Scr* into silk spheres. The results are expressed as the mean ± SD. * Indicates significance at p ≤ 0.05.

Both H2.1MS1KN and a blend of H2.1MS1:MS2KN proteins formed spheres, indicating that neither the KN peptide nor the addition of MS2KN protein impeded sphere formation. The morphology of the H2.1MS1KN and blend of H2.1MS1:MS2KN spheres was quite different (Figure 3A). Although both H2.1MS1KN and blended spheres indicated the spherical shape, the spherical form of the blended H2.1MS1:MS2KN spheres was more clearly distinguished. These spheres were more defined and separated. Additionally, based on the analysis of SEM images, it can be concluded that the H2.1MS1KN spheres exhibited lower stability. After two weeks of storage, they exhibited a less spherical shape and reduced structural distinction. Moreover, the mean size of spheres indicated by analysis of the SEM images was approximately 336.2 ± 20 nm (mean ± standard error of the mean) for H2.1MS1:MS2KN and 393.1 ± 29 nm for H2.1MS1KN spheres, while by using NanoSight analyzer 148 ± 12 nm and 190 ± 8 nm, respectively. The discrepancies in the obtained mean size of particles may result from difference in sample preparation and the experimenter’s subjective selection of larger, well-defined spheres during SEM image analysis. Since the mean ± standard error of the mean value did not show the particle size distribution, we presented the D90 obtained from the NanoSight analyzer in Figure 3B. Analysis indicated that 90% of the H2.1MS:MS2KN particles were of size 205 ± 30 nm or smaller, while H2.1MS1KN spheres 286 ± 50 nm (Figure 3B). Regardless of the measurement method, H2.1MS1:MS2KN particles were consistently smaller than H2.1MS1KN spheres, and analysis of D90 values indicated a significant difference in particle size.

Our previous results indicated that MS1- and MS2-based spheres differed in shape and size.^52^,^71^ The SEM picture analysis indicated that the MS2-based spheres were smaller (426 (±109) nm vs 246 (±66) nm for MS1 vs MS2), and the spherical morphology was more distinct compared with MS1-type particles.^52^ The observed differences were probably due to their higher beta-sheet content (crystallinity) and the presumed presence of beta-spirals in MS2 spheres, which could lead to the tight packing of the silk molecules into particles.^71^ Additionally, we performed the study for blended silk particles (MS1:MS2), indicating that 20% MS2 content in the MS1:MS2 spheres resulted in the smaller and more spherical in shape spheres formation comparing with MS1, however the content of beta-sheet in blended spheres was similar to the plain MS1 particles.^52^ The data indicated in this study are in agreement with our previous result in terms of the size and morphology of spheres; the addition of the MS2 component to MS1 silk influenced the characteristics of silk particles. On the other hand, the analyzed spheres also contained KN peptides, which could be critical for their physicochemical characterization. Indeed, the addition of poly-lysine molecules decreased the size of MS2 spheres from 246 (±66) nm to 182.7 nm (±6.4 nm).^52^,^68^ Moreover, Numata et al indicated that the size of plain MS1-type particles changed from 570 nm to 210 nm after adding poly-lysine peptide, and their size depended on the number of Lysine repeats.^79^ Thus, the size and morphology of both spheres presented in this study were probably modified by poly-lysine peptide and MS2 silk, resulting in more favorable characteristics for blended H2.1MS1:MS2KN particles.

The zeta potential of the H2.1MS1KN and H2.1MS1:MS2KN particles was similar. The average of ZP for H2.1MS1:MS2KN was 23 (± 4) mV and for H2.1MS1KN 23 (± 6) mV (Figure 3C). It was interesting that blended spheres indicated similar ZP to H2.1MS1KN spheres. The H2.1MS1KN particles are built of silk in which each molecule contains poly-lysine peptide (KN), while in the blended H2.1MS1:MS2KN spheres, only 20% of silk is functionalized with KN. It indicates that the number of positively charged residues in the silk particles is not a dominant factor for its overall ZP value, but rather their distribution and manner of packing the silk molecules into spheres. A similar ZP in both types of spheres is also associated with a comparable potential for loading nucleic acids. The loading efficiency of oligonucleotides was similar, approximately 53% for H2.1MS1KN and 56% for the H2.1MS1:MS2KN spheres (Figure 3D). There was an increase in a negatively charged oligonucleotide embedding into blended particles compared with plain H2.1MS1KN, but the difference was insignificant.

### The Cytotoxicity Analysis H2.1MS1KN and H2.1MS1:MS2KN Spheres

**Figure 4:**
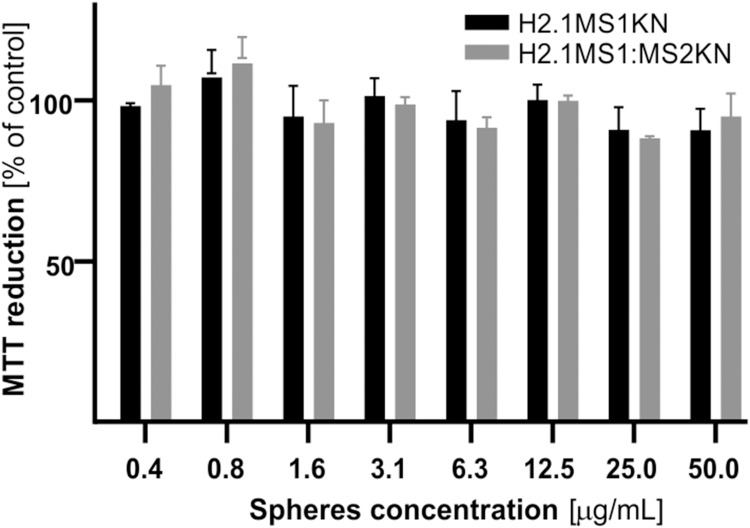
Cytotoxicity evaluation of silk spheres. SKOV3 ovary cancer cells were incubated with various concentrations of H2.1MS1KN and H2.1MS1:MS2KN silk spheres, and cell metabolic activity was assessed using the MTT assay. The percentage of MTT reduction was determined relative to untreated control cells. Data are presented as the mean of three independent experiments, with error bars representing the standard error of the mean.

Spheres made of H2.1MS1KN and a blend of H2.1MS1 and MS2KN2 silks were not cytotoxic when examined at a wide range of concentrations (Figure 4). There was no significant difference between H2.1MS1KN and H2.1MS1:MS2KN spheres in terms of cytotoxicity. As demonstrated in our previous study, plain MS2KN spheres unloaded with siRNA exhibited toxic properties when tested at a high concentration in contrast to MS2 particles, indicating potential cytotoxicity of KN peptide.^68^ Using only 20% of MS2KN protein in the H2.1MS1:MS2KN blend did not induce a similar effect. Interestingly, H2.1MS1KN particles were also not cytotoxic despite containing a high KN content. It may suggest that MS1-type protein may mask the cytotoxic effects of the KN peptide.

### The Binding Property of H2.1MS1KN and H2.1MS1:MS2KN Silk Spheres to HER2+ Cancer Cells

**Figure 5:**
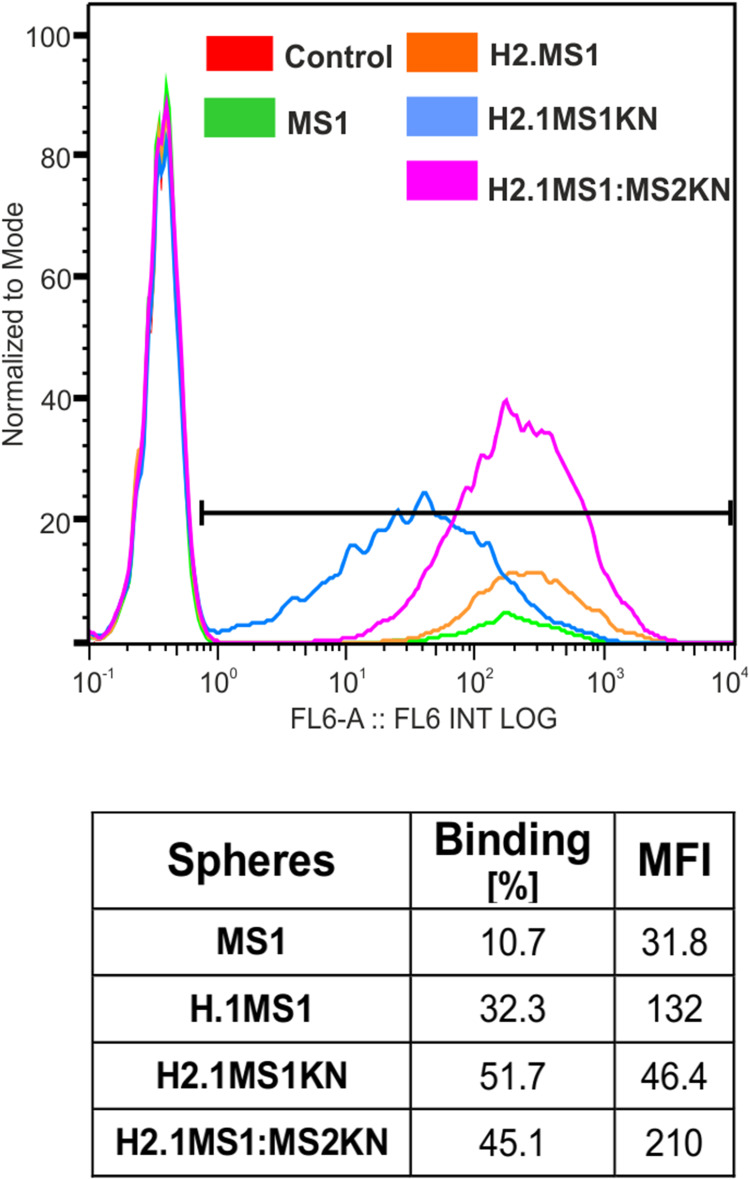
Flow cytometry analysis of sphere binding by HER+ cancer cells. The human SKOV3 ovary cancer cells were incubated for 1 h with Cy5-labeled H2.1MS1KN or H2.1MS1:MS2KN silk spheres and analyzed by flow cytometry.

To assess the binding property of functionalized spheres to HER2+ cancer cells, we incubated HER2-overexpressing SKOV3 ovarian cancer cells with fluorescently labeled (Cy5) spheres for 1 hour without transfection reagents. Flow cytometric analysis confirmed that the H2.1 peptide increased the binding of silk spheres to HER2-overexpressing cancer cells (Figure 5) Additionally, adding positively charged KN peptide increased the cell-binding potential of H2.1MS1KN and H2.1MS1:MS2KN particles and the percentage of cells that bound fluorescently labeled spheres were higher than controls (Figure 5). However, the MFI analysis indicated the considerable difference between cells that interact with the H2.1MS1KN and H2.1MS1:MS2KN spheres, the H2.1MS1:MS2KN being one significantly more accepted by cells (number of bound-spheres per cell).

### The Internalization of H2.1MS1KN and H2.1MS1:MS2KN Silk Spheres into HER2+ Cancer Cells

**Figure 6:**
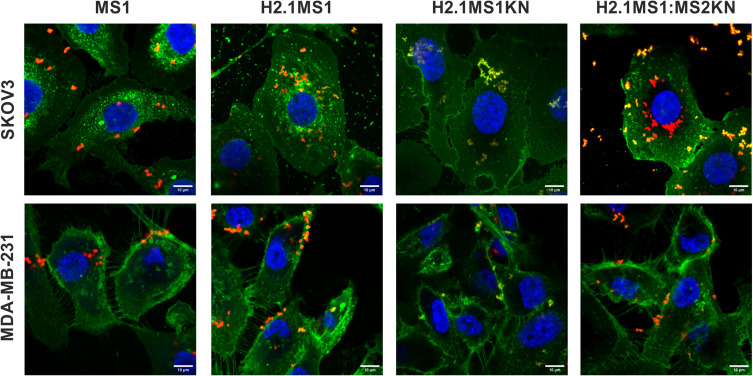
Confocal microscopy analysis of the cellular uptake of H2.1MS1KN, H2.1MS1:MS2KN and MS1 control silk spheres. The HER2-overexpressing human ovary cancer cells SKOV3, and HER2-negative human breast cancer cells MDA-MB-231 were incubated with silk particles for 4 h. FITC-Concanavalin A was used for staining cell surface carbohydrates (green), silk spheres were labeled with Cy5 (red), and nucleus was stained with DAPI (blue). Scale bar - 10 μm.

The flow cytometry analysis indicated unequal distribution of cells that bound of H2.1MS1KN vs H2.1MS1:MS2KN spheres suggesting different efficiency of their internalization. Thus, the cellular internalization of silk spheres into HER2- (MDA-MB-231) and HER2+ (SKOV3) cancer cells was analyzed using confocal microscopy (Figure 6). The singular MS1 particles (control) were visible inside the HER2- and HER2+ cancer cells. Some H2.1MS1KN spheres were internalized into HER2+ cancer cells and were present on the cell surface of HER2- cells; however, their fluorescent signal (red) was co-localized with FITC-labelled membrane structures. The H2.1MS1:MS2KN spheres were present in the cytosol of the HER2+ cancer cells at the highest quantity compared with other particles. Although H2.1MS1:MS2KN spheres indicated some binding to the HER2- MDA-MB-231 breast cancer cells, most of the particles remained on the cell surface and did not internalize (Figure 6).

We indicated previously that functionalization with the H2.1 peptide significantly increased the cellular internalization of silk spheres.^53^,^63^ The flow cytometry analysis suggested that further functionalization with KN peptide increased the cell-binding potential of silk spheres (Figure 5). The positively charged peptides, like poly-lysine or poly arginine, can penetrate the cell membrane, and they are often used for the functionalization of different types of nanoparticles to enable their cellular internalization.^80–83^ In our previous study, we indicated that MS2KN silk spheres (without a specific targeting peptide) were internalized by macrophages.^68^ However, the main macrophage’s function is phagocytosis of various molecules, including bacteria, viruses, cells, and others; thus, the internalization of the MS2KN particles could be related to such macrophage’s properties. In this study, we indicated that adding poly-lysine peptide caused distinct effects depending on sphere type. Indeed, there was more abounded number of the blended H2.1MS1:MS2KN particles in the cytoplasm of SKOV3 cancer cells than H2.1MS1 one, suggesting the synergistic effect of KN and H2.1 peptides. However, the H2.1MS1KN internalized at a much lower level into SKOV3 cells. Moreover, based on the CLSM pictures, it can be suggested that the silk particles stuck into the cell membrane (the co-localization of the fluorescent signals of spheres and cell membrane). Although the MTT assay did not indicate the influence of silk particles on cellular metabolism, the analysis of microscopic pictures may suggest the influence of H2.1MS1KN particles on cellular morphology. It was reported previously that poly-lysine, as a cationic polymer, could be toxic to cells when applied at a high concentration.^80^,^83^ Summarizing, the obtained data suggests that a high number of poly-lysine residues (again, each H2.1MS1KN silk molecule contains KN peptide) is not beneficial for cell interaction. Interestingly, some parts of the blended H2.1MS1:MS2KN particles also co-localized with the cell membrane, contrary to H2.1MS1 spheres (Figure 6). Probably, it was an intermediate state since a much larger number of them occurred in the cytoplasm.

### Analysis of the Gene Silencing Effect Exerted of siRNA Delivered Using H2.1MS1KN and H2.1MS1:MS2KN Spheres

**Figure 7:**
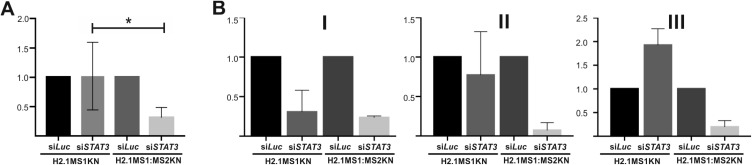
Analysis of *STAT3* mRNA level after treatment with si*STAT3* delivered by H2.1MS1KN and H2.1MS1:MS2KN spheres. (**A**) The HER2+ SKOV3 cancer cells were incubated for 48 h with H2.1MS1KN or H2.1MS1MS2KN spheres loaded with si*STAT3* (250nM) or si*Luc*(250 nM) and then qPCR analysis was performed. *STAT3* mRNA expression level was normalized to *GAPDH* expression. *Indicates significance at p ≤ 0.05. Results are expressed as means of six independent experiments and error bars show the standard error of the mean. (**B**) After being loaded with siRNA, the spheres were stored for (I) 1 week, II) 2 weeks, or III) 3 weeks and then used for *STAT3* silencing. Each experiment was performed once after indicated time of storage (I–III) in technical repetitions (mean ± SD). The mean value and the standard error of the mean. of the silencing effect of si*STAT3* delivered by H2.1MS1KN shown in 7A were calculated based on the results obtained for stored spheres from different time points.

The si*STAT3* (250 nM) and control si*Luc* (250 nM) were encapsulated into H2.1MS1KN and H2.1MS1:MS2KN silk spheres, applied on the SKOV3 HER2+ cancer cells, and the STAT3 silencing effect was determined by quantitative real-time PCR analysis. The six time repeated experiments indicated that H2.1MS1:MS2KN spheres delivered si*STAT3* more effectively than H2.1MS1KN particles, which caused significantly higher downregulation of STAT3 expression into SKOV3 cells (Figure 7A). The same preparation of silk spheres was used three times with a one-week interval, and the silencing effect of *STAT3* was repetitively achieved for si*RNA* delivered by H2.1MS1:MS2KN spheres (Figure 7B; I–III). The efficacy of H2.1MS1KN particles for delivering functional si*STAT3* decreased in time (Figure 7B; I–III). The experimental cycle was repeated, and the results were similar (data not shown). The reason for the loosing time-related potential of H2.1MS1KN spheres for delivery active siRNA was not examined in detail. However, it may be related to the unstable morphology of the spheres, releasing of siRNA, exposure more cytotoxic poly-lysine residues or combination of these. As the MTT study indicated that none silk particles modified the cellular metabolism, the cytotoxicity was not the direct reason for the lower performance of H2.1MS1KN particles. However, SEM image analysis indicated that the spheres’ instability may be responsible for the reduction in siRNA delivery efficiency of H2.1MS1KN over time. The unstable morphology of the spheres may contribute to the release of siRNA and inadequate interaction/recognition of the cells, decreasing the overall performance of H2.1MS1KN particles in siRNA delivery. It needs further detailed explanation; however, it was not within the scope of this study.

## Conclusion

We constructed an efficient, stable, and cell-specific delivery system for therapeutic siRNA molecules to HER2+ cancer cells using bioengineered silk spheres vehicles. The blended H2.1MS1:MS2KN spheres were effectively loaded with oligonucleotides and delivered si*STAT3* to HER2+ cancer cells that successfully silenced STAT3 expression. The blended H2.1MS1:MS2KN spheres are better choice than H2.1MS1KN particles due to i) higher efficiency of protein yields, ii) smaller size, iii) more defined spherical morphology, iv) higher HER2+ cell-binding potential, v) higher specific internalization into HER-overexpressing cancer cells, and finally vi) more efficient and stable delivery of functional siRNA. The superior property of the H2.1MS1:MS2KN spheres may be due to lower number of poly-lysine residues in the particles and stabilizing influence of MS2-type silk. H2.1MS1:MS2KN particles have emerged as a promising siRNA targeted delivery system with great potential for use in the treatment of HER2-overexpressing cancers. The reasonable next step is in vivo evaluation of the safety and efficiency of the developed DDS. In the future, the silk-based DDS that precisely delivers oligonucleotide therapeutics can be tested alongside other strategies to treat cancer, including chemo-, photodynamic, thermal, immune-, or hormone therapy. Nowadays, various DDSs, including silk-based ones, are being tested to fulfill the requirements of such strategies. Undoubtedly, targeted delivery systems are key to optimizing safe, side-effect-minimized treatment and will continue to evolve. Silk is very promising for these applications as a biocompatible and degradable material.

## References

[1] Kaur R, Bhardwaj A, Gupta S. Cancer treatment therapies: traditional to modern approaches to combat cancers. *Mol Biol Rep*. 2023;50(11):9663–9676. doi:10.1007/s11033-023-08809-3 37828275

[2] Cesur‐Ergün B, Demir‐Dora D. Gene therapy in cancer. *J Gene Med*. 2023;25(11):e3550. doi:10.1002/jgm.3550 37354071

[3] Chung SS, Giehl N, Wu Y, Vadgama JV. STAT3 activation in HER2-overexpressing breast cancer promotes epithelial-mesenchymal transition and cancer stem cell traits. *Int J Oncol*. 2014;44(2):403–411. doi:10.3892/ijo.2013.2195 24297508 PMC3898805

[4] Molenda S, Sikorska A, Florczak A, Lorenc P, Dams-Kozlowska H. Oligonucleotide-based therapeutics for STAT3 targeting in cancer—drug carriers matter. *Cancers*. 2023;15(23):5647. doi:10.3390/cancers15235647 38067351 PMC10705165

[5] Hossain DMS, Dos Santos C, Zhang Q, et al. Leukemia cell–targeted STAT3 silencing and TLR9 triggering generate systemic antitumor immunity. *Blood*. 2014;123(1):15–25. doi:10.1182/blood-2013-07-517987 24169824 PMC3879904

[6] Kunigal S, Lakka SS, Sodadasu PK, Estes N, Rao JS. Stat3-siRNA induces Fas-mediated apoptosis in vitro and in vivo in breast cancer. *Int J Oncol*. 2009;34(5):1209–1220. 19360334 PMC2668130

[7] Rahmati M, Johari B, Kadivar M, Rismani E, Mortazavi Y. Suppressing the metastatic properties of the breast cancer cells using STAT3 decoy oligodeoxynucleotides: a promising approach for eradication of cancer cells by differentiation therapy. *J Cell Physiol*. 2020;235(6):5429–5444. doi:10.1002/jcp.29431 31912904

[8] Moreira D, Adamus T, Zhao X, et al. STAT3 inhibition combined with CpG immunostimulation activates antitumor immunity to eradicate genetically distinct castration-resistant prostate cancers. *Clin Cancer Res*. 2018;24(23):5948–5962. doi:10.1158/1078-0432.CCR-18-1277 30337279 PMC6279477

[9] Pawlus MR, Wang L, Hu CJ. STAT3 and HIF1α cooperatively activate HIF1 target genes in MDA-MB-231 and RCC4 cells. *Oncogene*. 2014;33(13):1670–1679. doi:10.1038/onc.2013.115 23604114 PMC3868635

[10] McDaniel JM, Varley KE, Gertz J, et al. Genomic regulation of invasion by STAT3 in triple negative breast cancer. *Oncotarget*. 2017;8(5):8226–8238. doi:10.18632/oncotarget.14153 28030809 PMC5352396

[11] Paunovska K, Loughrey D, Dahlman JE. Drug delivery systems for RNA therapeutics. *Nat Rev Genet*. 2022;23(5):265–280. doi:10.1038/s41576-021-00439-4 34983972 PMC8724758

[12] Haupenthal J, Baehr C, Kiermayer S, Zeuzem S, Piiper A. Inhibition of RNAse A family enzymes prevents degradation and loss of silencing activity of siRNAs in serum. *Biochem Pharmacol*. 2006;71(5):702–710. doi:10.1016/j.bcp.2005.11.015 16376306

[13] Mittal M, Kumari A, Paul B, et al. Challenges and opportunities of gene therapy in cancer. *OBM Gene*. 2024;08(01):1–501. doi:10.21926/obm.genet.2401219

[14] Shaw BR, Dobrikov M, Wang X, et al. Reading, writing, and modulating genetic information with boranophosphate mimics of nucleotides, DNA, and RNA. *Ann N.Y Acad Sci*. 2003;1002(1):12–29. doi:10.1196/annals.1281.004 14751819

[15] Kurreck J. Design of antisense oligonucleotides stabilized by locked nucleic acids. *Nucleic Acids Res*. 2002;30(9):1911–1918. doi:10.1093/nar/30.9.1911 11972327 PMC113840

[16] Bramsen JB, Pakula MM, Hansen TB, et al. A screen of chemical modifications identifies position-specific modification by UNA to most potently reduce siRNA off-target effects. *Nucleic Acids Res*. 2010;38(17):5761–5773. doi:10.1093/nar/gkq341 20453030 PMC2943616

[17] Altuntaş E, Özkan B, Güngör S, Özsoy Y. Biopolymer-based nanogel approach in drug delivery: basic concept and current developments. *Pharmaceutics*. 2023;15(6):1644. doi:10.3390/pharmaceutics15061644 37376092 PMC10301308

[18] Zhang Y, Liu X, Li X. Development and Advantages Of Drug Delivery Systems. In: Teng L, Yang Z, Li C editors. *Drug Delivery to Tumors*. Springer Nature Singapore; 2025:35–60. doi:10.1007/978-981-19-8930-8_2.

[19] Nie Y, Li D, Peng Y, et al. Metal organic framework coated MnO2 nanosheets delivering doxorubicin and self-activated DNAzyme for chemo-gene combinatorial treatment of cancer. *Int J Pharm*. 2020;585:119513. doi:10.1016/j.ijpharm.2020.119513 32526334

[20] Castellino SM, Pei Q, Parsons SK, et al. Brentuximab vedotin with chemotherapy in pediatric high-risk Hodgkin’s lymphoma. *N Engl J Med*. 2022;387(18):1649–1660. doi:10.1056/NEJMoa2206660 36322844 PMC9945772

[21] Yu X, Liu S, Cheng Q, et al. Lipid‐modified aminoglycosides for mRNA delivery to the liver. *Adv Healthcare Mater*. 2020;9(7):1901487. doi:10.1002/adhm.201901487 PMC8152636 32108440

[22] Ezike TC, Okpala US, Onoja UL, et al. Advances in drug delivery systems, challenges and future directions. *Heliyon*. 2023;9(6):e17488. doi:10.1016/j.heliyon.2023.e17488 37416680 PMC10320272

[23] Yang H, Li Q, Chen X, et al. Targeting SOX13 inhibits assembly of respiratory chain supercomplexes to overcome ferroptosis resistance in gastric cancer. *Nat Commun*. 2024;15(1):4296. doi:10.1038/s41467-024-48307-z 38769295 PMC11106335

[24] Loke YH, Jayakrishnan A, Mod Razif MRF, et al. A comprehensive review of challenges in oral drug delivery systems and recent advancements in innovative design strategies. *CPD*. 2025;31(5):360–376. doi:10.2174/0113816128338560240923073357 39390835

[25] Milewska S, Niemirowicz-Laskowska K, Siemiaszko G, Nowicki P, Wilczewska AZ, Car H. Current trends and challenges in pharmacoeconomic aspects of nanocarriers as drug delivery systems for cancer treatment. *IJN*. 2021;16:6593–6644. doi:10.2147/IJN.S323831 34611400 PMC8487283

[26] Patra JK, Das G, Fraceto LF, et al. Nano based drug delivery systems: recent developments and future prospects. *J Nanobiotechnol*. 2018;16(1):71. doi:10.1186/s12951-018-0392-8 PMC6145203 30231877

[27] Moskowitz AJ, Shah G, Schöder H, et al. Phase II trial of pembrolizumab plus gemcitabine, vinorelbine, and liposomal doxorubicin as second-line therapy for relapsed or refractory classical Hodgkin lymphoma. *JCO*. 2021;39(28):3109–3117. doi:10.1200/JCO.21.01056 PMC9851707 34170745

[28] Schmid P, Adams S, Rugo HS, et al. Atezolizumab and Nab-paclitaxel in advanced triple-negative breast cancer. *N Engl J Med*. 2018;379(22):2108–2121. doi:10.1056/NEJMoa1809615 30345906

[29] Rodríguez F, Caruana P, De La Fuente N, et al. Nano-based approved pharmaceuticals for cancer treatment: present and future challenges. *Biomolecules*. 2022;12(6):784. doi:10.3390/biom12060784 35740909 PMC9221343

[30] Linxweiler H, Thiesen J, Krämer I. Physicochemical stability of nab-paclitaxel (Pazenir) infusion dispersions in original glass vials and EVA infusion bags. *Pharmaceutics*. 2024;16(11):1372. doi:10.3390/pharmaceutics16111372 39598496 PMC11597360

[31] Torres-Vanegas JD, Cruz JC, Reyes LH. Delivery systems for nucleic acids and proteins: barriers, cell capture pathways and nanocarriers. *Pharmaceutics*. 2021;13(3):428. doi:10.3390/pharmaceutics13030428 33809969 PMC8004853

[32] Dume B, Licarete E, Banciu M. Advancing cancer treatments: the role of oligonucleotide-based therapies in driving progress. *Mol Ther Nucleic Acids*. 2024;35(3):102256. doi:10.1016/j.omtn.2024.102256 39045515 PMC11264197

[33] Pan J, Ruan W, Qin M, et al. Intradermal delivery of STAT3 siRNA to treat melanoma via dissolving microneedles. *Sci Rep*. 2018;8(1):1117. doi:10.1038/s41598-018-19463-2 29348670 PMC5773564

[34] Morgan E, Wupperfeld D, Morales D, Reich N. Shape matters: gold nanoparticle shape impacts the biological activity of siRNA delivery. *Bioconjugate Chem*. 2019;30(3):853–860. doi:10.1021/acs.bioconjchem.9b00004 30735028

[35] Seleci DA, Seleci M, Jochums A, Walter JG, Stahl F, Scheper T. Aptamer mediated niosomal drug delivery. *RSC Adv*. 2016;6(91):87910–87918. doi:10.1039/C6RA19525C

[36] Jiang S, Wang X, Zhang Z, et al. CD20 monoclonal antibody targeted nanoscale drug delivery system for doxorubicin chemotherapy: an in vitro study of cell lysis of CD20-positive Raji cells. *Int J Nanomed*. 2016;11:5505–5518. doi:10.2147/IJN.S115428 PMC5098746 27843311

[37] Florczak A, Deptuch T, Kucharczyk K, Dams-Kozlowska H. Systemic and local silk-based drug delivery systems for cancer therapy. *Cancers*. 2021;13(21):5389. doi:10.3390/cancers13215389 34771557 PMC8582423

[38] Ramezaniaghdam M, Nahdi ND, Reski R. Recombinant spider silk: promises and bottlenecks. *Front Bioeng Biotechnol*. 2022;10:835637. doi:10.3389/fbioe.2022.835637 35350182 PMC8957953

[39] Agostini E, Winter G, Engert J. Water-based preparation of spider silk films as drug delivery matrices. *J Control Release*. 2015;213:134–141. doi:10.1016/j.jconrel.2015.06.025 26100366

[40] Herold HM, Döbl A, Wohlrab S, Humenik M, Scheibel T. Designed spider silk-based drug carrier for redox- or pH-triggered drug release. *Biomacromolecules*. 2020;21(12):4904–4912. doi:10.1021/acs.biomac.0c01138 33249826

[41] Schacht K, Vogt J, Scheibel T. Foams made of engineered recombinant spider silk proteins as 3D scaffolds for cell growth. *ACS Biomater Sci Eng*. 2016;2(4):517–525. doi:10.1021/acsbiomaterials.5b00483 33465855

[42] Deptuch T, Dams-Kozlowska H. Silk materials functionalized via genetic engineering for biomedical applications. *Materials*. 2017;10(12):1417. doi:10.3390/ma10121417 29231863 PMC5744352

[43] Tokareva O, Michalczechen‐Lacerda VA, Rech EL, Kaplan DL. Recombinant DNA production of spider silk proteins. *Microb Biotechnol*. 2013;6(6):651–663. doi:10.1111/1751-7915.12081 24119078 PMC3815454

[44] Trossmann VT, Scheibel T. Design of recombinant spider silk proteins for cell type specific binding. *Adv Healthcare Mater*. 2023;12(9):2202660. doi:10.1002/adhm.202202660 PMC11468868 36565209

[45] Kucharczyk K, Florczak A, Kaminska A, et al. MMPs-responsive silk spheres for controlled drug release within tumor microenvironment. *Int J Biol Macromol*. 2024;269:132016. doi:10.1016/j.ijbiomac.2024.132016 38697442

[46] Huang T, Kumari S, Herold H, et al. Enhanced antibacterial activity of se nanoparticles upon coating with recombinant spider silk protein eADF4(κ16). *Int J Nanomed*. 2020;15:4275–4288. doi:10.2147/IJN.S255833 PMC7306472 32606677

[47] Lammel A, Schwab M, Hofer M, Winter G, Scheibel T. Recombinant spider silk particles as drug delivery vehicles. *Biomaterials*. 2011;32(8):2233–2240. doi:10.1016/j.biomaterials.2010.11.060 21186052

[48] Mulinti P, Shreffler J, Hasan R, Dea M, Brooks AE. Infection responsive smart delivery of antibiotics using recombinant spider silk nanospheres. *Pharmaceutics*. 2021;13(9):1358. doi:10.3390/pharmaceutics13091358 34575434 PMC8467577

[49] Numata K, Reagan MR, Goldstein RH, Rosenblatt M, Kaplan DL. Spider silk-based gene carriers for tumor cell-specific delivery. *Bioconjugate Chem*. 2011;22(8):1605–1610. doi:10.1021/bc200170u PMC3157559 21739966

[50] Kucharczyk K, Kaczmarek K, Jozefczak A, Slachcinski M, Mackiewicz A, Dams-Kozlowska H. Hyperthermia treatment of cancer cells by the application of targeted silk/iron oxide composite spheres. *Mater Sci Eng C*. 2021;120:111654. doi:10.1016/j.msec.2020.111654 33545822

[51] Kucharczyk K, Florczak A, Deptuch T, et al. Drug affinity and targeted delivery: double functionalization of silk spheres for controlled doxorubicin delivery into Her2-positive cancer cells. *J Nanobiotechnol*. 2020;18(1):56. doi:10.1186/s12951-020-00609-2 PMC7106823 32228620

[52] Florczak A, Jastrzebska K, Mackiewicz A, Dams-Kozlowska H. Blending two bioengineered spider silks to develop cancer targeting spheres. *J Mat Chem B*. 2017;5(16):3000–3011. doi:10.1039/C7TB00233E 32263992

[53] Florczak A, Mackiewicz A, Dams-Kozlowska H. Functionalized spider silk spheres as drug carriers for targeted cancer THERAPY. *Biomacromolecules*. 2014;15(8):2971–2981. doi:10.1021/bm500591p 24963985

[54] Van Der Geer P, Hunter T, Lindberg RA. Receptor protein-tyrosine kinases and their signal transduction pathways. *Ann Rev Cell Biol*. 1994;10(1):251–337. doi:10.1146/annurev.cb.10.110194.001343 7888178

[55] Swain SM, Shastry M, Hamilton E. Targeting HER2-positive breast cancer: advances and future directions. *Nat Rev Drug Discov*. 2023;22(2):101–126. doi:10.1038/s41573-022-00579-0 36344672 PMC9640784

[56] Baselga J. Clinical trials of Herceptin^®^ (trastuzumab). *Eur J Cancer*. 2001;37:18–24. doi:10.1016/S0959-8049(00)00404-4 11342196

[57] Ryan Q, Ibrahim A, Cohen MH, et al. FDA drug approval summary: lapatinib in combination with capecitabine for previously treated metastatic breast cancer that overexpresses HER-2. *oncologist*. 2008;13(10):1114–1119. doi:10.1634/theoncologist.2008-0816 18849320

[58] Mahlknecht G, Maron R, Mancini M, Schechter B, Sela M, Yarden Y. Aptamer to ErbB-2/HER2 enhances degradation of the target and inhibits tumorigenic growth. *Proc Natl Acad Sci*. 2013;110(20):8170–8175. doi:10.1073/pnas.1302594110 23630281 PMC3657787

[59] Kim B, Shin J, Wu J, et al. Engineering peptide-targeted liposomal nanoparticles optimized for improved selectivity for HER2-positive breast cancer cells to achieve enhanced in vivo efficacy. *J Control Release*. 2020;322:530–541. doi:10.1016/j.jconrel.2020.04.010 32276005 PMC7932755

[60] Quinn Z, Mao W, Xia Y, John R, Wan Y. Conferring receptors on recipient cells with extracellular vesicles for targeted drug delivery. *Bioact Mater*. 2021;6(3):749–756. doi:10.1016/j.bioactmat.2020.09.016 33024896 PMC7522541

[61] Hashemi-Moghaddam H, Zavareh S, Karimpour S, Madanchi H. Evaluation of molecularly imprinted polymer based on HER2 epitope for targeted drug delivery in ovarian cancer mouse model. *React Funct Polym*. 2017;121:82–90. doi:10.1016/j.reactfunctpolym.2017.10.025

[62] Satpathy M, Wang L, Zielinski RJ, et al. Targeted drug delivery and image-guided therapy of heterogeneous ovarian cancer using HER2-targeted theranostic nanoparticles. *Theranostics*. 2019;9(3):778–795. doi:10.7150/thno.29964 30809308 PMC6376473

[63] Florczak A, Mackiewicz A, Dams-Kozlowska H. Cellular uptake, intracellular distribution and degradation of Her2-targeting silk nanospheres. *Int J Nanomed*. 2019;14:6855–6865. doi:10.2147/IJN.S217854 PMC6716583 32021156

[64] Florczak A, Deptuch T, Lewandowska A, et al. Functionalized silk spheres selectively and effectively deliver a cytotoxic drug to targeted cancer cells in vivo. *J Nanobiotechnol*. 2020;18(1):177. doi:10.1186/s12951-020-00734-y PMC7709326 33261651

[65] Deptuch T, Florczak A, Lewandowska A, et al. Ms1-type bioengineered spider silk nanoparticles do not exhibit toxicity in an in vivo mouse model. *Nanomedicine*. 2021;16(18):1553–1565. doi:10.2217/nnm-2021-0029 34165326

[66] Deptuch T, Penderecka K, Kaczmarek M, Molenda S, Dams-Kozlowska H. In vivo study of the immune response to bioengineered spider silk spheres. *Sci Rep*. 2022;12(1):13480. doi:10.1038/s41598-022-17637-7 35931709 PMC9356052

[67] Deptuch T, Kucharczyk K, Florczak A, Dams‐Kozlowska H. Endotoxin reduction from biotech silk material inhibits the production of anti‐silk antibodies in mice. *J Biomed Mater Res Part A*. 2024;112(3):463–472. doi:10.1002/jbm.a.37644 37941467

[68] Kozlowska AK, Florczak A, Smialek M, et al. Functionalized bioengineered spider silk spheres improve nuclease resistance and activity of oligonucleotide therapeutics providing a strategy for cancer treatment. *Acta Biomater*. 2017;59:221–233. doi:10.1016/j.actbio.2017.07.014 28694238 PMC5942204

[69] Dams‐Kozlowska H, Majer A, Tomasiewicz P, Lozinska J, Kaplan DL, Mackiewicz A. Purification and cytotoxicity of tag‐free bioengineered spider silk proteins. *J Biomed Mater Res Part A*. 2013;101A(2):456–464. doi:10.1002/jbm.a.34353 PMC3494781 22865581

[70] Jastrzebska K, Felcyn E, Kozak M, et al. The method of purifying bioengineered spider silk determines the silk sphere properties. *Sci Rep*. 2016;6:28106. doi:10.1038/srep28106 27312998 PMC4911573

[71] Jastrzebska K, Florczak A, Kucharczyk K, et al. Delivery of chemotherapeutics using spheres made of bioengineered spider silks derived from MaSp1 and MaSp2 proteins. *Nanomedicine*. 2018;13(4):439–454. doi:10.2217/nnm-2017-0276 29338625 PMC5810845

[72] Bunce NAC, White RP, Shewry PR. Variation in estimates of molecular weights of cereal prolamins by SDS-PAGE. *J Cereal Sci*. 1985;3(2):131–142. doi:10.1016/S0733-5210(85)80023-0

[73] Smart JE, Bonner J. Selective dissociation of histones from chromatin by sodium deoxycholate. *J Mol Biol*. 1971;58(3):651–659. doi:10.1016/0022-2836(71)90030-1 5104701

[74] Rodriguez-Collazo P, Leuba SH, Zlatanova J. Robust methods for purification of histones from cultured mammalian cells with the preservation of their native modifications. *Nucleic Acids Res*. 2009;37(11):e81–e81. doi:10.1093/nar/gkp273 19443446 PMC2699528

[75] Shechter D, Dormann HL, Allis CD, Hake SB. Extraction, purification and analysis of histones. *Nature Protocols*. 2007;2(6):1445–1457. doi:10.1038/nprot.2007.202 17545981

[76] Vaara M. Agents that increase the permeability of the outer membrane. *Microbiol Rev*. 1992;56(3):395–411. doi:10.1128/mr.56.3.395-411.1992 1406489 PMC372877

[77] Tan Z, Shi Y, Xing B, Hou Y, Cui J, Jia S. The antimicrobial effects and mechanism of ε-poly-lysine against Staphylococcus aureus. *Bioresources Bioprocess*. 2019;6(1):11. doi:10.1186/s40643-019-0246-8

[78] Švedienė J, Novickij V, Žalnėravičius R, et al. Antimicrobial activity of L-Lysine and Poly-L-Lysine with pulsed electric fields. *Appl Sci*. 2021;11(6):2708. doi:10.3390/app11062708

[79] Numata K, Subramanian B, Currie HA, Kaplan DL. Bioengineered silk protein-based gene delivery systems. *Biomaterials*. 2009;30(29):5775–5784. doi:10.1016/j.biomaterials.2009.06.028 19577803 PMC2732109

[80] Granier F, Marie S, Al Amir Dache Z, et al. Assessment of dendrigrafts of poly- l -lysine cytotoxicity and cell penetration in cancer cells. *ACS Appl Polymer Mater*. 2022;4(2):908–919. doi:10.1021/acsapm.1c01354

[81] Riveros AL, Eggeling C, Riquelme S, et al. Improving cell penetration of gold nanorods by using an amphipathic arginine rich peptide. *Int J Nanomed*. 2020;15:1837–1851. doi:10.2147/IJN.S237820 PMC7090188 32256063

[82] Zhang H, Men K, Pan C, et al. Treatment of colon cancer by degradable rrPPC nano-conjugates delivered STAT3 siRNA. *Int J Nanomed*. 2020;15:9875–9890. doi:10.2147/IJN.S277845 PMC7732178 33324056

[83] Mohamed A, Korzhikov-Vlakh V, Zhang N, et al. Effect of Poly(L-lysine) and heparin coatings on the surface of polyester-based particles on prednisolone release and biocompatibility. *Pharmaceutics*. 2021;13(6):801. doi:10.3390/pharmaceutics13060801 34072016 PMC8229182

